# Fat deposition of parotid glands

**DOI:** 10.5935/1808-8694.20130031

**Published:** 2015-11-02

**Authors:** Davi Sousa Garcia, Ivo Bussoloti Filho

**Affiliations:** a Otorhinolaryngologist (Fellow in Rhinology - Otorhinolaryngology Department - Santa Casa de São Paulo); b PhD in Otorhinolaryngology. Adjunct Professor and Director - Department of Otorhinolaryngology - Santa Casa de São Paulo. Otorhinolaryngology Department - Irmandade da Santa Casa de Misericórdia de São Paulo

**Keywords:** fats, magnetic resonance imaging, parotid gland

## Abstract

Parotid gland parenchyma histology may be altered by local or systemic, pathological or non-pathological conditions.

**Objective:**

This paper aims to highlight the fatty degeneration of the parotid gland, a not well-known non-pathological condition.

**Method:**

In a retrospective study, we collected a series of 6 patients which presented a swelling of the parotid, but even after extensive research, a diagnosis was not reached.

**Results:**

Through the retrospective analysis of records we found fatty degeneration of the parotid in an MRI scan of all patients.

**Conclusion:**

This condition, despite being physiological and expected with aging, may be related to clinical and radiological swelling of these glands.

## INTRODUCTION

Parotid gland parenchyma is subject to changes in its histological characteristics because of local or systemic conditions. Hypertriglyceridemia may be associated with fat infiltration in the parotid without inflammatory activity^1^. The drop in ovarian hormones after ovariectomy in rats showed fat degeneration in the parotid glands^2^. In Sjögren's syndrome, there is parenchyma heterogeneity because of adipose degeneration and lymphocytic infiltration^3^. In patients with the Human Immunodeficiency Virus (HIV), there may be parotid gland enlargement and, histologically, besides the fat infiltration, one may see lymphocytic aggregates, lymphoepithelial cysts or lymphadenopa-thy^4^. Moreover, in alcoholics and diabetics, there is a reduction in the rate of fat tissue in the stroma^5^. On the other hand, studies with rats have shown an increase in lipids and degeneration of secretory granules associated with an age-related decline in the secretory activities of the parotid glands^6^, without it representing a disease process.

In the stomatology ward of our institution, we had patients in whom we saw an increase in the volume of the parotid glands, eventually associated with complaints resembling those of a Sjögren's syndrome, but after broad diagnostic investigation, such condition was ruled out, based on current diagnostic criteria (European-American Consensus, 2002)^7^. By the same token, any other local or systemic conditions, such as sarcoidosis^8^ and lymphoma^9^, which would explain that situation, have just been discarded by means of pertaining propaedeutic. Nonetheless, during the diagnostic investigation process through an MRI, we noticed that a common point to them is the finding of liposubstitution of the parotid glands.

Therefore, this study aimed at informing about this pathology, which makes patients with this little-known pathology seek physicians.

## METHOD

This is a historical cross-sectional cohort study, carried out with the charts of 6 patients seen in the stomatology ward of our institution, between February of 2009 and January of 2012.

**Figure 1:**
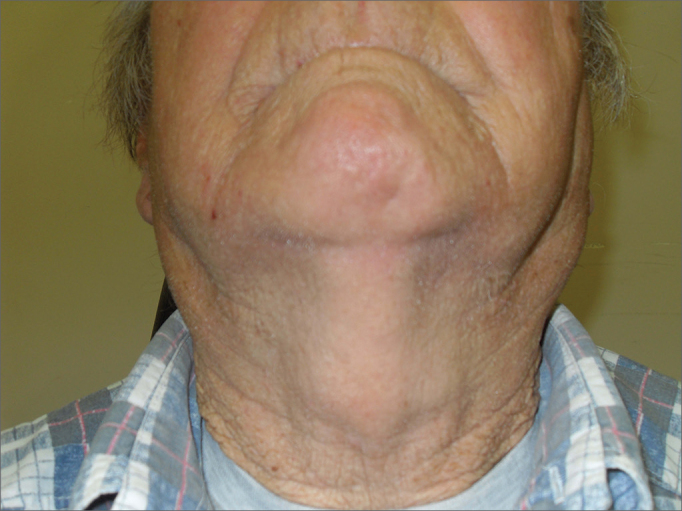
Patient with bilateral parotid enlargement and liposubstitution seen upon MRI.

The inclusion criterion was patients who had clinical signs of volumetric increase of the parotid glands, without any local or systemic conditions that would justify them, after the pertaining diagnostic investigation, by means of an image exam, laboratorial and/or anatomic-pathological tests (Figure 1). In these cases, our investigation encompasses a complete blood count, antiSSB, anti-nucleus factor, rheumatoid factor, HIV, anti-HCV, Schirmer test, minor salivary gland biopsy, neck MRI, scintigraphy and, in selected cases: sialography.

As for inclusion criterion, we defined the cases which were truly unilateral through the MRI.

Since it is a retrospective study based on information from patients' charts, without patient identification, there was no need for informed consent forms.

The project was approved by the Clinical Board and by the Ethics in Research Committee of the institution, under protocol # 49.444/12.

## RESULTS

Within the time frame considered (February of 2009 through January of 2012), we admitted 483 new patients in our ward, of whom 64 (13.25%) had enlarged parotids as their main complaint, and six (1.24%) fit the inclusion criteria.

**Figure 2:**
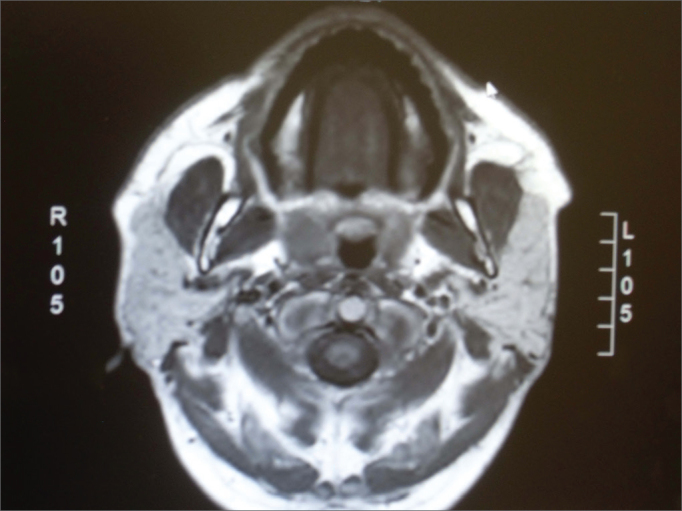
Axial section in T1 without contrast, showing hypersignal from the parotids.

**Figure 3:**
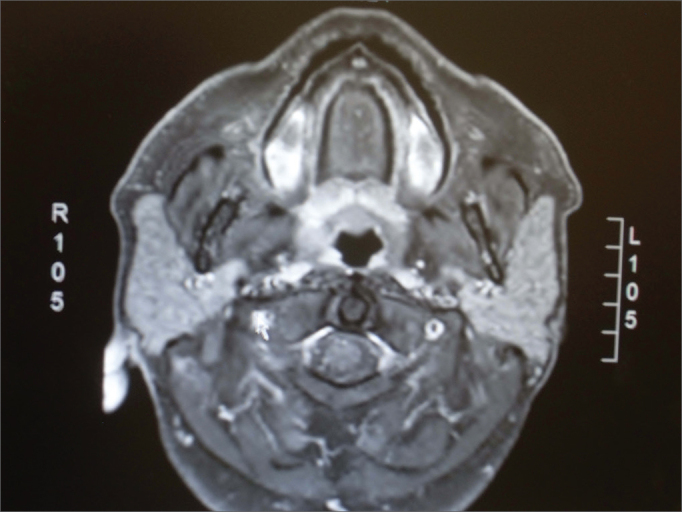
Axial section in T1 with contrast and fat suppression, showing hyposignal in the areas in which fat was suppressed.

Upon MRI, the fat deposits on the parotids was seen by a T1 hypersignal; in fat suppression sequences, the suppressed sites are identified by hyposignal (T1 with contrast and T2)^10^ (Figures 2 and 3).

The results obtained were plotted on the table below (Table 1).

**Table 1:** Information from the patients who fit the inclusion criteria.

1^st^ visit	Gender	Age of onset	Complaint	Alcoholism	Smoking	Physical exam	MRI
02/21/09	M	58	Parotid bulging for 8 months, recurrent, bilateral and painful, with xerostomy	No	No	Mild bilateral enlargement of the parotids, larger on the left, fibroelastic	Bilateral parotid volume enlargement, with fat substitution signs, without nodules
10/06/09	M	68	Bilateral parotid bulging for 1 year, without salivary complaints	No	No	Bilateral salivary gland bulging, especially the parotids	Parotid parenchyma replacement for fat, with some small lymph nodes
10/06/09	F	46	Bilateral parotid bulging for 1 year	No	Yes	Diffuse parotid enlargement, fibroelastic	Liposubstitution and parotid volume enlargement
05/10/11	F	33	Right parotid bulging for 3 years, mild xerostomy	No	No	Right-side parotid volume enlargement	Volume enlargement and parotid liposubstitution
11/29/11	M	55	Parotid bulging for 4 months, with xerostomy	Yes	No	Mild bilateral and fibro-elastic parotid enlargement	Diffuse parotid enlargement, with liposubstitution
01/31/12	F	46	Recurrent right side, painful bulging for 2 years	No	No	Right-side parotid enlargement, mildly painful upon palpation	Parotid liposubstitution and small intraparotid lymph nodes

The data collected point to three male patients and three females; therefore, there was no gender distinction. Age of onset varied between 33 and 68 years, with a mean age of 51 (±12.06) years. The trait was recurrent in two patients (33.33%), being constant in the others. In all the patients, the parotid consistency was firm/fibroelastic, without irregularities or inflammatory signs, although mildly painful in one patient. We stress that xerostomy was complained by three patients (50%), confirmed upon clinical exam; notwithstanding, these patients were submitted to parotid gland scintigraphy, without any change in the tests. As far as habits are concerned, one patient (16.67%) reported being an ex-smoker and one patient (16.67%) reported mild alcohol consumption. In all the patients we found signs of liposubstitution of the parotid glands upon the MRI in both parotids, although in two cases they were unilateral.

We stress that the 64 cases of parotid enlargement in our ward had the following diagnosis: Sjögren's syndrome (15), repeating parotiditis of childhood (13), sialadenitis (7), infectious (7), tumoral (5), intraparotid cyst (4), HIV (2), lymphoma (2), alcoholic (1), sarcoi-dosis (1), liposubstitution (7). Of these four cases in which we found parotid liposubstitution, one case was taken off the study since the MRI showed unilateral changes.

## DISCUSSION

Parotid liposubstitution is a frequent radiologic finding; however, the literature does not have papers correlating idiopathic parotid bulging with its presence. It is known that this is a natural physiological process, like aging^6^ (we stress that the mean age of our sample was 51 years); however, subject to variations in accordance with the presence of subjacent clinical conditions^1^, ^2^, ^3^, ^4^, ^5^. In fact, the parotid liposubstitution with the Sjögren's syndrome is known, after having been demonstrated by Niemela et al.^11^, in a study involving 26 patients with the primary form of the disease, and changes (nodules or adipose degeneration in the parenchyma) were seen in 81% of the cases.

On the other hand, our series was made up of individuals without local or systemic diseases, exhaustively investigated, they presented parotid liposubstitution as a common denominator, which leads us to think that, in some cases, this is not a mere histological transformation as per expected with the gland aging, having a clinical and radiological manifestation of parotid enlargement. Parotid bulging was recurrent in two patients, which is not expected for a supposedly slow and gradual process. And finally, there are issues which still cannot be cleared up, especially since this is a theme with very little presence in the medical literature.

## CONCLUSION

Parotid liposubstitution, although a physiological process - expected with aging, may be associated with a bilateral enlargement of the glands in individuals who do not have systemic or local causes for such condition. However, some issues related to this association, remain open, and may be investigated in new studies.
